# Effects of diets containing fish oils or fish oil concentrates with high cetoleic acid content on the circulating cholesterol concentration in rodents. A systematic review and meta-analysis

**DOI:** 10.1017/S0007114523002118

**Published:** 2024-02-28

**Authors:** Margrete Mjaatveit, Helle Oldernes, Oddrun Anita Gudbrandsen

**Affiliations:** Department of Clinical Medicine, University of Bergen, Haukeland University Hospital, Bergen, 5021, Norway

**Keywords:** Rat, Mouse, Long-chain MUFA, Pelagic fish

## Abstract

Hypercholesterolaemia is a major risk factor for CVD. Fish intake is associated with lower risk of CVD, whereas supplementation with *n*-3 long-chain PUFA (LC-PUFA) has little effect on the cholesterol concentration. We therefore investigated if cetoleic acid (CA), a long-chain MUFA (LC-MUFA) found especially in pelagic fish species, could lower the circulating total cholesterol (TC) concentration in rodents. A systematic literature search was performed using the databases PubMed, Web of Science and Embase, structured around the population (rodents), intervention (CA-rich fish oils or concentrates), comparator (diets not containing CA) and the primary outcome (circulating TC). Articles were assessed for risk of bias using the SYRCLE’s tool. A meta-analysis was conducted in Review Manager v. 5.4.1 (the Cochrane Collaboration) to determine the effectiveness of consuming diets containing CA-rich fish oils or concentrates on the circulating TC concentration. Twelve articles were included in the systematic review and meta-analysis, with data from 288 rodents. Consumption of CA-rich fish oils and concentrates resulted in a significantly lower circulating TC concentration relative to comparator groups (mean difference −0·65 mmol/l, 95 % CI (−0·93, −0·37), *P* < 0·00001), with high statistical heterogeneity (*I* ^2^ = 87 %). The risk of bias is unclear since few of the entries in the SYRCLE’s tool were addressed. To conclude, intake of CA-rich fish oils and concentrates prevents high cholesterol concentration in rodents and should be further investigated as functional dietary ingredients or supplements to reduce the risk for developing CVD in humans.

CVD is the leading cause of disease burden globally with around 523 million cases and 18·6 million deaths in 2019^(1)^. An elevated cholesterol concentration is a significant risk factor for the development CVD^(2,3)^ and is associated with the long-term risk of coronary heart disease and all-cause mortality^(4,5)^, therefore, lowering the serum cholesterol concentration is a central approach for reducing the CVD risk^(6)^. The CVD primary prevention strategy includes lifestyle modifications and use of lipid-lowering drugs^(7)^, and non-pharmacological treatment of hypercholesterolaemia including desirable changes in dietary habits is the recommended first approach^(7,8)^. Several observational studies have demonstrated that a high fish consumption is associated with a lower risk of CVD^(9,10)^, and consuming fish at least once per week significantly reduces the risk of cardiovascular mortality^(11)^. Marine fish and seafood are rich dietary sources for the *n*-3 long-chain PUFA (LC-PUFA) EPA (C20:5*n*-3) and DHA (C22:6*n*-3), and the beneficial effects of marine foods have traditionally been ascribed to these fatty acids^(12)^. However, fish oils and concentrates with high EPA and DHA contents do not affect the cholesterol concentration when consumed by humans^(13–16)^ and lower the cholesterol concentration in rats and mice only when given in very high doses corresponding to 10–40 % of energy as EPA and DHA^(17)^. Oils isolated from certain fish species feeding in the pelagic zone, such as herring, are characterised by a relatively low content of EPA and DHA but contain high amounts of the long-chain MUFA (LC-MUFA) cetoleic acid (CA, C22:1*n*-11). Despite its relatively low *n*-3 LC-PUFA content, consumption of herring fillet increased the HDL-cholesterol concentration in healthy adults^(18,19)^ and resulted in a lower TC concentration in hypercholesterolaemic mice^(20)^ and in Wistar rats fed a high fat diet^(21)^.

The knowledge regarding the possible health effects of LC-MUFA such as CA is limited compared with the vast amount of information gathered from studies investigating the effects of the well-known *n*-3 LC-PUFAs. Little is known about the metabolism of CA after consumption in humans and other animals, and we know little about its health effects and the possible mechanisms of action behind such effects. Studies using tritium-labelled CA in minks (*Mustela vison*) and grey seals (*Halichoerus grypus*) show that CA is chain-shortened by oxidation, mainly to C18:1 but also to C16:1 and C20:1, and is further oxidised in mitochondria to acetyl groups to be used for *de novo* fatty acid synthesis^(22)^.

The main aim of this systematic review and meta-analysis was to investigate the effect of intake of diets containing CA, either *as is* or as part of fish oils or fish oil concentrates, on serum/plasma TC concentration in rodents. The secondary aim was to provide a narrative synthesis of the findings from the included articles structured around the population (rodents), the intervention (source of CA, dose, duration), the comparator (type of oil) and composition of diets (regular rodent diet, high-fat diet, high-carbohydrate diet, diet added cholesterol alone or in combination with cholate) on the circulating concentrations of TC, HDL-cholesterol and LDL-cholesterol, the hepatic content of TC, the daily faecal excretion of TC and total bile acids and on any relevant proteins involved in the cholesterol metabolism in the liver. Also, information regarding the rodents’ hepatic contents of CA, EPA and DHA were collected as a measure of uptake of these fatty acids and for discussion of CA’s metabolism and possible mechanisms of action. Findings from this systematic review and meta-analysis will provide important knowledge regarding possible health effects of fish oils and fish oil concentrates that have a high content of CA but low or no content of EPA and DHA, and may be important for the design of future studies in humans and animals targeting the effects of fish oils on cholesterol metabolism.

## Methods

### Protocol and registration

The review protocol can be viewed at the International prospective register of systematic reviews (PROSPERO) website (https://www.crd.york.ac.uk/PROSPERO), with registration number CRD42023398867.

### Search strategy

A comprehensive literature search was carried out in accordance with the Preferred Reporting Items for systematic reviews and Meta-Analyses (PRISMA) guidelines^(23)^, using the three electronic databases PubMed (https://pubmed.ncbi.nlm.nih.gov/), Web of Science (https://clarivate.com/products/web-of-science/) and Embase (http://www.elsevier.com/online-tools/embase). The search was conducted in May 2023.

The following search terms were combined to identify relevant articles on the effects of diets containing CA *per SE* or fish oils or fish oil concentrates with a high content of CA on serum/plasma cholesterol concentration in rodents: (“cetoleic acid” OR “docosenoic acid” OR “C22:1" OR “LCMUFA” OR “LC MUFA” OR “LC-MUFA” OR “long-chain monounsaturated fatty acid*” OR “herring” OR “capelin” OR “tobis” OR “sand eel” OR “sandeel” OR “redfish” OR “saury” OR “mackerel” OR “sprat” OR “pollock”) AND (“oil” OR “oils”) AND (“rat” OR “rats” OR “mouse” OR “mice” OR “chinchilla” OR “guinea pig” OR “hamster” OR “rodent”). Since dietary intake of zooplankton such as copepods is the main source for CA in fish, we have included names of fish that feed in the pelagic zone and are relevant for human nutrition, to broaden the search. The primary outcome for the present systematic review, i.e. circulating TC concentration, was not included in the search since this outcome was not necessarily the primary outcome in the eligible articles.

The reference lists of the reviewed articles were checked manually to identify relevant studies. We searched for reviews on the topic using PubMed, Web of Science and Embase, and we searched Prospero (https://www.crd.york.ac.uk/PROSPERO/) to avoid overlap with similar systematic reviews.

### Selection criteria

The search strategy was composed from the PICO (Participants, Interventions, Comparisons and Outcomes) concept framework^(24)^ and was based on keywords for each of the PICO categories. Eligibility criteria were (1) population: only articles from intervention studies using rodents were included, but articles using neonate rodents, pregnant rodents, surgical animal models, rodents with chemically induced diseases or metabolic changes were excluded; (2) intervention: the intervention must comprise CA or non-hydrogenated fish oil or fish oil concentrate with high CA content as part of a diet with regular protein content (14–25 wt %) for a period of minimum 5 d, thus excluding oils/fatty acids administered by oral gavage or by injection or in drinking water, single-dose administration and short time studies (<5 d); (3) comparison: the study design must include a control group fed an isocaloric diet with no or very low content of CA or long-chain *n*-3 PUFAs, contain sufficient amounts of essential fatty acids and have comparable contents of macronutrients, and (4) outcomes; the main outcome was circulating TC concentration at endpoint, the secondary outcomes were HDL-cholesterol, LDL-cholesterol, hepatic TC, daily faecal excretion of TC and bile acids, and any information related to cholesterol metabolism to elucidate mechanisms behind observed effect on markers, e.g. mRNA level, protein concentrations and activity of enzymes involved in cholesterol metabolism and adiposity at endpoint. Review articles, protocols, abstracts, posters and grey literature were not included. We did not exclude articles on the basis of the articles’ publication year or language during the identification process. Articles were manually removed if there were no English full texts available.

All three authors individually performed the search and evaluated the articles. The retrieved articles from PubMed, Web of Science and Embase were transferred to the free web-tool Rayyan (www.rayyan.ai) where duplicates were identified by the software, and then removed manually. The screening was performed in two phases. The initial screening was based on the title and abstract, followed by a full-text screening of the eligible publications for final inclusion. In each phase, all authors independently assessed each article and any discrepancies were resolved through discussion, and articles were included or excluded based on the eligibility criteria.

### Data extraction and criteria appraisal

Data were extracted from article text, tables and figures based on the categories defined for each of the PICO categories described above. The extracted data included rodent species, strain, sex, bodyweight and age at baseline, experimental and comparator groups, number of animals per group, description of the intervention diets (fish species, fish oil or concentrate, percentage of fat from fish oils/concentrates and other fats in the intervention diet, any additions of cholesterol and cholate to the diets) and the comparator diets (percentage of fat from different sources, any additions of cholesterol and cholate to the diets), diet availability (e.g. *ad libitum* or pair-feeding), the duration of the intervention period and the prandial state at the time of euthanisation or collection of relevant blood samples. The CA concentration in the fish oils and fish oil concentrates was used for the estimation of the CA contents in the diets. Data collected for outcome measurements included the main outcome, i.e. circulating TC concentration which was described in all included articles, and the secondary outcomes were HDL-cholesterol, LDL-cholesterol, hepatic TC content, daily faecal excretion of TC and bile acids and hepatic mRNA level/protein content/activity of CYP7A1 (cholesterol 7 *α*-hydroxylase, the rate-determining enzyme for the conversion of cholesterol to bile acids), HMG-CoA reductase (the rate-determining enzyme for the hepatic synthesis of cholesterol), the LDL-receptor which binds to the apolipoproteins apoB100 and apoE, sterol O-acyltransferase 2 (SOAT2, also known as acyl-CoA:cholesterol acyltransferase) which catalyse the esterification of cholesterol and the sterol regulatory element-binding protein 2 which regulates the cholesterol homeostasis by stimulating transcription of sterol-regulated genes, and any other measured proteins that are relevant for cholesterol metabolism. In addition, information on dietary intake and adiposity at endpoint were extracted.

The results from the included articles were defined as statistically significant where *P* < 0·05. For articles that included multiple dietary groups, only data from the relevant fish oil or fish oil concentrate and the comparator groups were extracted and are included in this review; however, the statistical testing in the articles may not be ideal for the experimental groups that were selected for the present review. Examples of this may be that the comparator group selected for the present paper may be different from the designated control group in the original articles, and the statistical testing may be against a control group that is not identical to our chosen comparator group.

The data extraction was completed independently by the authors. When inconsistencies were observed in the articles, e.g. missing data or information or suspicion of possible errors, the corresponding authors were contacted by e-mail or through ResearchGate (https://www.researchgate.net).

An evaluation of the extracted data revealed that study designs in the included articles were highly heterogeneous with regard to, e.g. rodent models, diet composition and the dose of CA, therefore a descriptive approach in addition to a meta-analysis was performed.

### Assessments of risk of bias and study quality

All reviewed articles were assessed for risk of bias by using the SYRCLE’s risk of bias tool for animal studies^(25)^, which is an adapted version of Cochrane’s risk of bias tool for clinical randomised trials^(26)^. The SYRCLE tool contains ten entries, with detailed signalling questions to help uncover potential bias. The questions were answered with Yes (low risk of bias), No (high risk of bias) or Unclear (unclear risk of bias). The quality of the included articles was evaluated by using a combination of the Collaborative Approach to Meta-Analysis and Review of Animal Data from Experimental Studies checklist^(27)^ and items from the Animal Research: Reporting of *In Vivo* Experiments (ARRIVE) 2·0 guidelines^(28)^. We selected these items to incorporate matters subjectively viewed as necessary for evaluating the overall quality of the studies, and we avoided items already covered by the SYRCLE’s risk of bias tool. The articles were scored with one point for reporting the required information and zero points when information is missing, with a total score of maximum 10 points, with 1–3 points regarded as low quality, 4–7 points regarded as medium quality, and 8–10 points regarded as high quality. All authors independently assessed each article for risk of bias and study quality, and any discrepancies were resolved through discussion.

### Statistical analyses

A meta-analysis was conducted using Review Manager v. 5.4.1^(29)^ (provided by the Cochrane Collaboration). The effect of consuming diets containing CA on circulating TC concentration (the primary outcome) from all included articles was meta-analysed by comparison to a relevant control diet as the comparator. The mean and standard deviation for endpoint TC concentration and the number of rodents were recorded for intervention and comparator groups. Standard deviation was calculated from the standard error of the mean when not provided in the articles. In articles presenting the TC concentration using graphs, we did our best to estimate each group’s mean and spread. For articles with more than one experiment, the experiments were analysed individually. For TC concentrations presented as mg/dl, these were converted to mmol/l by multiplying with 0·02586. Data were treated as continuous measures, and the intervention and comparator groups were compared using the random effects inverse-variance model as described by DerSimonian and Laird^(30)^. The statistical heterogeneity between studies was evaluated and is expressed as measures of Cochran’s Q (*χ* ^2^ test) and *I* ^2^. Sensitivity analyses were conducted through a leave-one-out analysis to evaluate the effect of confounding factors on the robustness. The comparisons with the highest positive and negative effect sizes, the studies with the highest risk of bias or lowest quality of evidence, were excluded sequentially from the meta-analysis. A funnel plot was produced to evaluate the publication bias. Subgroup analyses were conducted for fish oils *v* concentrates, and for comparison diets containing vegetable fat *v* animal fat. The results from the main meta-analysis and the subgroup analyses were visualised as forest plots. *P* < 0·05 was considered statistically significant. The secondary outcomes are reviewed narratively.

## Results

### Search results and study characteristics

**Fig. 1.**
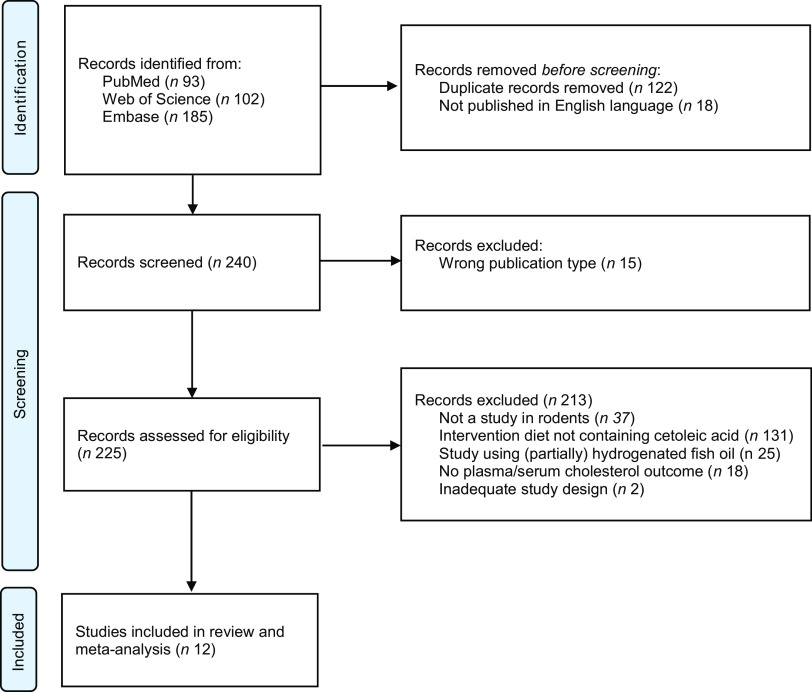
PRISMA flowchart of literature search via databases, showing the selection of studies for inclusion in the systematic review and meta-analysis.

We identified 258 potentially relevant articles, and of these, eighteen were excluded since they were not published in English language and fifteen were the wrong publication type (reviews and conference abstracts). Of the 225 articles assessed for eligibility, 186 were excluded based on the title or abstract, and twenty-seven were excluded due to ineligibility issues after full-text screening. Twelve articles, comprising a total of sixteen feeding experiments, were found eligible and were included in this systematic review and meta-analysis, comprising a total of 288 rodents. Of the reviewed articles, four used rats and eight used mice. Eligible articles using rodents other than rats and mice were not identified. The PRISMA 2020 flow diagram^(23)^ gives an overview of the selection process (Fig. 1), and the study characteristics are presented in Table 1 and Supplemental Table 1. The oldest included study was published in 1986, and the newest study was published in May of 2023. Both male and female rodents were used in the included articles; nine articles reported the use of solely male rats^(31–34)^ or male mice^(35–39)^, two articles included both female and male rats^(40)^ or mice^(41)^ and one article used solely female mice^(42)^. The age of the rats was reported to be 4 weeks^(40)^ or 10–12 weeks^(34)^ old, and the mice’ age were 6 weeks^(36–39)^, 7 weeks^(35)^ or 8 weeks^(42)^ at the initiation of the intervention period. Based on the stated bodyweights for the Wistar rats where age were not declared, we used the Charles River growth chart for male Wistar rats^(43)^ to estimate the age at baseline to be approximately 5–7 weeks for male Wistar rats weighing 175–185 g^(31)^, 5–8 weeks for male Wistar rats weighing 195–225 g^(32)^ and 4–6 weeks for male Wistar rats weighing 120 g^(33)^. One article gave no information on age or bodyweight of animals at the intervention start^(41)^. The durations of the intervention periods were between 3^(31,32)^ and 18^(35)^ weeks. The numbers of animals in each experimental group were stated in all articles, with group sizes between six and twelve rodents. The blood samples for analyses of TC, HDL-cholesterol and LDL-cholesterol were collected at the time of euthanasia in most articles^(31–34,36,38–40,42)^, whereas retro-orbital blood was collected within 1 week before euthanasia for analyses of lipids in three of the included articles^(35,37,41)^.

**Table 1.** Study characteristics and outcomes

First author Year Reference number	Sex and rodent strain. BW and age at intervention start	Description of the experimental groups, the number of animals in each group (*n*), and a brief description of experimental diets (proteins, simple carbohydrates, maltodextrin, fats and added cholesterol, shown as wt %)	Total cholesterol (TC), HDL-cholesterol and LDL-cholesterol concentrations in circulation, daily faecal excretion of cholesterol and total bile acids, hepatic proteins relevant for cholesterol metabolism in CA fed groups relative to comparator group and hepatic contents of CA, GA, EPA and DHA
Mounie *et al*. 1986 ^(33)^	Male Wistar rats BW: 120 g Age: N/A	1. Intervention diet with 6 % herring oil, *n* 10 2. Comparator diet with 6 % corn oil, *n* 10 Diet ingredients: 18 % casein, 30 % sucrose	Plasma TC: lower for the herring oil group HDL-cholesterol, LDL-cholesterol, liver TC, faecal cholesterol and TBA, liver CYP7A1, HMG-CoA reductase, LDL-receptor, SOAT-2, liver fatty acids: N/A
Dolphin *et al*. 1988 ^(40)^	Exp. 1: Male LA/N *cp/cp* rats BW: N/A Age: 4 weeks Exp. 2: Female LA/N *cp/cp* rats BW: N/A Age: 4 weeks	Both experiments: 1. Intervention diet: a normal low fat laboratory chow supplemented with 9·1 % redfish oil, *n* 6 2. Comparator diet: a normal low fat laboratory chow supplemented with 9·1 % olive oil, *n* 6 Diet ingredients: N/A	Exp. 1: Serum TC: NS HDL-cholesterol and LDL-cholesterol: lower for redfish oil group Liver TC, faecal cholesterol and TBA, liver CYP7A1, HMG-CoA reductase, LDL-receptor, SOAT-2, liver fatty acids: N/A Exp. 2: Serum TC and LDL-cholesterol: NS HDL-cholesterol: lower for redfish oil group Liver TC, faecal cholesterol and TBA, liver CYP7A1, HMG-CoA reductase, LDL-receptor, SOAT-2, liver fatty acids: N/A
Halvorsen *et al*. 1995 ^(31)^	Male Wistar rats BW: 175–185 g Age: N/A	1. Intervention diet with 6·5 % of LC-MUFA concentrate, 13 % lard and 1·5 % soybean oil, *n* 6 2. Comparator diet with 19·5 % lard and 1·5 % soybean oil, *n* 6 Diet ingredients: 20 % casein, 20 % sucrose	Plasma TC and liver TC: NS Fatty acids in liver-PL; CA and EPA: NS, DHA: higher in MUFA-group, GA: N/A HDL-cholesterol, LDL-cholesterol, faecal cholesterol and TBA, liver CYP7A1, HMG-CoA reductase, LDL-receptor, SOAT-2: N/A
Halvorsen *et al*. 2001 ^(32)^	Male Wistar rats BW: 195–225 g Age: N/A	1. Intervention diet with 6·5 % of LC-MUFA concentrate, 13 % lard and 1·5 % soybean oil, *n* 6 2. Comparator diet with 19·5 % lard and 1·5 % soybean oil, *n* 6 Diet ingredients: 20 % casein, 20 % sucrose	Plasma TC: NS Fatty acids in liver-PL; CA and GA: N/A, EPA and DHA: NS HDL-cholesterol, LDL-cholesterol, liver TC, faecal cholesterol and TBA, liver CYP7A1, HMG-CoA reductase, LDL-receptor, SOAT-2,: N/A
Yang *et al*. 2011 ^(37)^	Male C57BL/6J mice BW: ∼25 g Age: 6 weeks	1. Intervention diet with 15 % pollock oil, 17 % lard and 3·2 % soybean oil, *n* 10 2. Comparator diet with 32 % lard and 3·2 % soybean oil, *n* 10 Diet ingredients: 25·8 % casein, 8·9 % sucrose, 16·2 % maltodextrin	Plasma TC, LDL-cholesterol and liver TC: lower in pollock oil group HDL-cholesterol and liver ApoA mRNA: NS Liver HMG-CoA reductase, SREBP2 and ApoB mRNA: lower in pollock oil group Fatty acids in liver; CA, EPA and DHA: higher in pollock oil group, GA: N/A Faecal cholesterol and TBA, liver CYP7A1, LDL-receptor and SOAT-2: N/A
Yang *et al*. 2011 ^(38)^	Male C57BL/6J mice BW: ∼24 g Age: 6 weeks	1. Intervention diet with 5 % MUFA concentrate, 27 % lard and 3·2 % soybean oil, *n* 10 2. Comparator diet with 32 % lard and 3·2 % soybean oil, *n* 10 Diet ingredients: 25·8 % casein, 8·9 % sucrose, 16·2 % maltodextrin	Plasma TC and LDL-cholesterol: lower in MUFA concentrate group HDL-cholesterol: NS Fatty acids in liver; CA, EPA and DHA: higher in MUFA concentrate group, GA: N/A Liver TC, faecal cholesterol and TBA, liver CYP7A1, HMG-CoA reductase, LDL-receptor and SOAT-2: N/A
Yang *et al*. 2011 ^(39)^	Exp. 1: Male KK-A^y^ mice BW: ∼30 g Age: 6 weeks Exp. 2: Male C57BL/6J mice BW: ∼20–24 g Age: 6 weeks	Exp. 1: 1. Intervention diet with 10 % saury oil, *n* 10 2. Comparator diet with 10 % soybean oil, *n* 10 Diet ingredients: 20 % casein, 10 % sucrose Exp. 2: 1. Intervention diet with 10 % saury oil, 22 % lard and 3·2 % soybean oil, *n* 10 2. Comparator diet with 32 % lard and 3·2 % soybean oil, *n* 10 Diet ingredients: 25·8 % casein, 8·9 % sucrose, 16·2 % maltodextrin	Exp. 1: Plasma TC and LDL-cholesterol: lower in saury oil group HDL-cholesterol: N/A Liver TC, faecal cholesterol and TBA, liver CYP7A1, HMG-CoA reductase, LDL-receptor and SOAT-2, and liver fatty acids: N/A Exp. 2: Plasma TC and LDL-cholesterol: lower in saury oil group HDL-cholesterol: N/A Liver TC, faecal cholesterol and TBA, liver CYP7A1, HMG-CoA reductase, LDL-receptor and SOAT-2: N/A Fatty acids in liver; ΣC22:1, EPA and DHA higher in saury oil group, ΣC20:1 NS
Yang *et al*. 2013 ^(36)^	Male KK-A^y^ mice BW: ∼30 g Age: 6 weeks	1. Intervention diet with 4 % LC-MUFA concentrate and 3 % soybean oil, *n* 10 2. Comparator diet with 7 % soybean oil, *n* 10 Diet ingredients: 20 % casein, 10 % sucrose	Plasma TC: lower in LC-MUFA group Liver TC: NS Fatty acids in liver; GA and CA: higher, DHA: lower in LC-MUFA group, EPA: NS HDL-cholesterol, LDL-cholesterol, faecal cholesterol and TBA, liver CYP7A1, HMG-CoA reductase, LDL-receptor and SOAT-2: N/A
Yang *et al*. 2015 ^(35)^	Exp. 1: Male C57BL/6J mice BW: ∼23 g Age: 7 weeks Exp. 2: Male C57BL/6J mice BW: ∼23 g Age: 7 weeks	Exp. 1: 1. Intervention diet with 10 % saury oil, 22 % lard and 3·2 % soybean oil, *n* 10 2. Comparator diet with 32 % lard and 3·2 % soybean oil, *n* 10 Diet ingredients: 25·8 % casein, 8·9 % sucrose, 16·2 % maltodextrin Exp. 2: 1. Intervention diet with 12 % LC-MUFA concentrate, 20 % lard and 3·2 % soybean oil, *n* 10 2. Comparator diet with 32 % lard and 3·2 % soybean oil, *n* 10 Diet ingredients: 25·8 % casein, 8·9 % sucrose, 16·2 % maltodextrin	Exp. 1: Plasma TC, HDL-cholesterol, and liver TC: NS Non-HDL-cholesterol: lower in saury oil group Fatty acids in liver; CA, GA and Σn-3 PUFA (individual n-3 PUFA levels not presented): higher in saury oil group LDL-cholesterol, faecal cholesterol and TBA, liver CYP7A1, HMG-CoA reductase, LDL-receptor and SOAT-2: N/A Exp. 2: Plasma TC and non-HDL-cholesterol: lower in LC-MUFA group HDL-cholesterol and liver TC: NS Fatty acids in liver; CA, GA and DHA: higher in LC-MUFA group, EPA: NS LDL-cholesterol, faecal cholesterol and TBA, liver CYP7A1, HMG-CoA reductase, LDL-receptor and SOAT-2: N/A
Yang *et al*. 2016 ^(41)^	Exp. 1: Female LDLr^−/−^mice BW: N/A Age: N/A Exp. 2: Male apoE^-/^- mice BW: N/A Age: N/A	Exp. 1: 1. Intervention diet with 2 % LC-MUFA concentrate and 19 % milk fat, *n* 12 2. Comparator diet with 21 % milk fat, *n* 12 Diet ingredients: 19·5 % casein, 34·1 % sucrose, 0·15 % cholesterol Exp. 2: 1. Intervention diet with 5·2 % LC-MUFA-concentrate and 14·8 % milk fat, *n* 10 2. Comparator diet with 20 % milk fat, *n* 10 Diet ingredients: 19·5 % casein, 10 % sucrose, 10 % maltodextrin, 0·15 % cholesterol	Exp. 1 Plasma TC, HDL-cholesterol and LDL-cholesterol, and liver TC: NS Hepatic CYP7A1 mRNA: higher in LC-MUFA group Fatty acids in liver; CA, GA, EPA and DHA: higher in LC-MUFA group Faecal cholesterol and TBA, liver HMG-CoA reductase, LDL-receptor and SOAT-2: N/A Exp. 2: Plasma TC and HDL-cholesterol: NS LDL-cholesterol: lower in LC-MUFA group Hepatic CYP7A1 mRNA: higher in LC-MUFA group Liver TC, faecal cholesterol and TBA, liver HMG-CoA reductase, LDL-receptor and SOAT-2, liver fatty acids: N/A
Yang *et al*. 2017 ^(42)^	Female LDLr^−/−^ mice BW: N/A Age: 8 weeks	1. Intervention diet with 5·1 % CA-concentrate and 15·9 % milk fat, *n* 12 2. Comparator diet with 21 % milk fat, *n* 12 Diet ingredients: 19·5 % casein, 34·1 % sucrose, 0·15 % cholesterol	Plasma TC, HDL-cholesterol, LDL-cholesterol and liver TC: NS Fatty acids in liver; CA and GA: higher in LC-MUFA group, EPA and DHA: NS Faecal cholesterol and TBA, liver CYP7A1, HMG-CoA reductase, LDL-receptor and SOAT-2: N/A
Østbye *et al*. 2023 ^(34)^	Male Zucker fa/fa rats BW: 426 ± 16 g Age: 10–12 weeks	1. Intervention diet with 7 % sandeel oil and 5 % soybean oil*, n* 6 2. Comparator diet with 12 % soybean oil*, n* 6 Diet ingredients: 23·2 % casein, 10 % sucrose	Serum TC and HDL-cholesterol: NS Fatty acids in liver; CA and GA: N/A, EPA and DHA: higher in sandeel group LDL-cholesterol, liver TC, faecal cholesterol and TBA, liver CYP7A1, HMG-CoA reductase, LDL-receptor and SOAT-2: N/A

Apo, apolipoprotein; CA, cetoleic acid; CYP7A1, cholesterol 7 *α*-hydroxylase; GA, gadoleic acid; N/A, data not available; NS, not statistically significant; SOAT-2, sterol O-acyltransferase 2; SREBP2, sterol regulatory element-binding protein 2; TBA, total bile acids; TC, total cholesterol.

Blood was sampled from non-fasted animals in seven articles^(31,32,34,36,38,39,42)^, from fasting animals in four articles^(33,35,40,41)^, whereas one article gave no information of the prandial state of the animals at the time of blood sampling^(37)^. The experiments in the included articles were designed with *ad libitum* access to feed^(31–35,38,40)^ or pair feeding^(39)^, whereas four articles gave no information on whether feed was freely available or controlled^(36,37,41,42)^. The details for fasting conditions at blood sampling and the feed availability are presented in Supplemental Table 1.

### Assessments of risk of bias and quality of evidence

The SYRCLE’s risk of bias tool^(25)^ was used to assess the risk of bias in the reviewed articles, and results are presented in Supplemental Table 2. The use of *sequence generation* (#1) for reducing selection bias was not reported in any of the reviewed articles, and all articles were therefore graded with ‘Unclear’. Body weight and age were defined as the *baseline characteristics* (#2) that were compared between groups, and five of the articles^(34–38)^ included information that the animals were similar at baseline and were graded with ‘Yes’. The remaining seven articles did not did include a baseline comparison of animals, thus resulting in an unclear risk of bias. The risk of bias for the *allocation concealment* (#3) was unclear for all articles. The use of *random housing* (#4) was not reported in any of the articles; however, all articles received ‘Yes’, i.e. a low risk of bias, based on the signalling question ‘Is it unlikely that the outcome or the outcome measurement was influenced by not randomly housing the animals?’. All articles were graded as ‘unclear’ for the use of *blinding* in relation to *performance bias* (#5) and *detection bias* (#7) since the articles did not provide information whether caregivers and investigators had any knowledge on which experimental diets given to each animal. All articles were graded as having an unclear risk of bias for the *random outcome assessment* (#6), based on lack of relevant information. When scoring the articles for *incomplete outcome data* (#8), defined as the main outcome in the present study, all articles received ‘Yes’, i.e. a low risk of bias, as all the expected data were included. For the last two entries, *selective outcome reporting* (#9) and *other sources of bias* (#10), we graded all articles as having an unclear risk of bias. To summarise, the included articles were scored with between two or three ‘Yes’, with the rest of the scores being ‘Unclear’. Since few of the entries in the SYRCLE’s tool were addressed, we conclude that the risk of bias was unclear for the included articles.

All included articles were assessed for the quality of evidence for the primary outcomes (online Supplemental Table 3). All articles were peer reviewed (#1), included information of the animal model and strain used (#2), stated the sex of the experimental animals (#3) and gave details on the statistical method (#7) and descriptive statistics with a measure of variability (#8) for the serum/plasma TC concentration. Nine of the articles provided some information about housing and husbandry conditions, whereas none of the articles described any actions to improve animal welfare of the experimental animals (#4). For item #5, i.e. description of the procedures, we checked for information on when the blood was sampled, any use of anaesthesia when blood was sampled for TC analysis, and if information regarding the prandial condition of the animals were provided. All articles described when blood was collected for TC analysis, but one article did not provide information on prandial status^(37)^ and four articles gave no information regarding any use of anaesthesia at the time of sampling^(32,33,41,42)^. All articles described the analysis of serum/plasma TC, either by referring to the name and brand of assays and kits used or by describing the protocol (#6). Compliance with animal welfare regulations (#9) was declared in all articles except the four oldest articles^(31–33,40)^. Statement of potential conflict of interests (#10) was declared in six articles^(34–37,41,42)^. The overall quality was high for the included articles, with a mean score of 8·4 (range 7·0–9·75).

### Details on the rodent models

The included papers used the following rodent models: Wistar rats^(31–33)^, corpulent LA/N *cp/cp* rats^(40)^ obese Zucker *fa/fa* rats^(34)^, C57BL/6J mice^(35,37–39)^, KK-*A* ^*y*^ obese mice^(36,39)^, LDL receptor deficient mice (LDLr^-/^-)^(41,42)^ and apolipoprotein E null mice (apoE^-/^-)^(41)^ (Table 1).

The Wistar rat and the C57BL/6J mouse are regarded as being normocholesterolemic when fed a regular diet. The LA/N *cp/cp* rat is obese, hyperphagic and moderately hypercholesterolaemic due to markedly elevated VLDL and HDL levels^(44)^. The obese Zucker *fa/fa* rat spontaneously develops metabolic abnormalities including elevated concentrations of serum LDL, HDL and VLDL cholesterol^(45)^. The apoE^-/^- mouse^(46)^ and the LDLr^-/^- mouse^(47)^ are hypercholesterolaemic. The KK mouse carrying the yellow obese gene *A* ^*y*^ (KK-*A* ^*y*^) is obese and develops type 2 diabetes^(48)^.

### Details on the designs of the diets

The intervention diets were added fish oil or a fish oil concentrate containing CA, and none of the included experiments used diets supplemented with pure CA. The examined fish oils included herring oil^(33)^, redfish oil^(40)^, saury oil^(35,39)^, pollock oil^(37)^ and sandeel oil^(34)^, and the concentrates were described as LC-MUFA concentrate^(31,32,35,36,38,41)^ or CA concentrate^(42)^. The CA in the concentrates was stated to be in the form of an ethyl ester in five of the articles^(31,32,35,38,42)^, whereas two articles gave no information on whether CA was given as a free fatty acid, an esterified form or if it was otherwise prepared^(36,41)^. The CA content in oils or in the lipid fraction of prepared diets was described in all included articles except Mounie *et al.* ^(33)^ who reported the total C22:1 content in the fish oil (online Supplemental Table 4). The estimated CA content in the intervention diets shows great variation, spanning from the lowest content of 0·70 and up to 4·78 g of CA per 100 g diet. The dietary contents of the long-chain *n*-3 PUFAs EPA and DHA also showed great variation, with the highest levels in diets containing fish oils (EPA + DHA, range 0·66–2·74 g/100 g diet) and no or very low levels in diets with added LC-MUFA or CA concentrates (EPA + DHA, range 0–0·11 g/100 g diet). CA was not detected in any of the comparator diets; however, comparator diets containing lard or milk fat contained minute amounts of long-chain *n*-3 LC-PUFA (EPA + DHA, range 0·02–0·13 g/100 g diet). All diets contained the precursor for EPA and DHA, *α*-linoleic acid (ALA, C18:3*n*-3) in the range 0·05–0·59 g/100 g intervention diet and 0·07–0·83 g/100 g comparator diet.

Cholesterol was added to intervention and comparator diets in Yang 2016^(41)^ and in Yang 2017^(42)^; in both articles, the cholesterol supplementation was 0·15 g/100 g diet. The plant oils and the fish oil concentrates do not contain cholesterol, whereas fish oils, lard and milk fat contain cholesterol. The estimated cholesterol content in the diets containing fish oil or animal fats was between 0·01 and 0·20 mg/100 g diet in both intervention diets and comparator diets, and in the range 0·01 to 0·13 mg/100 g diet for diets not added cholesterol (online Supplemental Table 4). Cholate, which reduces the cholesterol catabolism in the liver by inhibiting the production of bile acids through down-regulation of hepatic CYP7A1 activity, was not added to any of the diets. In none of the experiments were cholesterol added solely the intervention diet or to the comparator diet in order to balance the cholesterol content between the diets.

All reviewed articles included a relevant comparator group that was fed a diet similar to the intervention diet of interest, with the exception of the lipid component. The dietary composition was in all other aspects similar between intervention and comparator diets and was described in detail in all included articles except in Dolphin *et al*.^(40)^ who used an unspecified normal low fat laboratory chow but provided no description of the composition. All included studies, with the possible exception of the Dolphin study^(40)^, used semi-purified diets with casein as the sole protein source in both intervention and comparator diets, with a dietary raw protein content between 15 and 25 wt %^(31–39,41,42)^. The comparator diets contained a variety of fat sources; either olive oil^(40)^, soybean oil^(34,36,39)^, corn oil^(33)^ or milk fat^(41,42)^ as the sole source of dietary fat, or a combination of lard and soybean oil^(31,32,35,37–39)^ or of milk fat and corn oil^(41)^. The dietary fat content ranged from a regular content of fat (≤7 wt % fat) to high-fat diets (≥30 wt % fat). The amounts of proteins, fats and simple carbohydrates were similar between the intervention diet and the comparator diet within each experiment in all articles.

### Cholesterol metabolism

The effect of consuming CA-rich fish oils and fish oil concentrates on the circulating TC concentration was described in all included articles, and the effects on HDL-cholesterol, LDL-cholesterol and hepatic TC concentration as well as the gene expressions of enzymes relevant for cholesterol metabolism were reported in several articles (Table 1). The faecal excretion of TC and bile acids was not measured in any of the included articles. The findings in the reviewed articles are presented in detail below.

### Effects of fish oils on markers for cholesterol metabolism

The serum TC concentration was not affected in male or female LA/N *cp/cp* rats fed a normal low fat laboratory chow supplemented with 9·1 % redfish oil when compared with a corresponding diet supplemented with olive oil^(40)^. However, the HDL-cholesterol concentration was lower in both male and female LA/N *cp/cp* rats, and the LDL-cholesterol concentration was lower in male LA/N *cp/cp* rats fed redfish oil diet when compared with the respective comparator groups^(40)^. The plasma TC concentration was lower in male Wistar rats fed a diet with regular fat content and a moderately high content of simple carbohydrates added 6 % herring oil compared with a corresponding diet with 6 % corn oil; in this experiment, HDL-cholesterol and LDL-cholesterol were not quantified^(33)^. The serum TC and HDL-cholesterol concentrations were not affected (LDL-cholesterol was not measured) in Zucker *fa/fa* rats fed a diet with 7 % sandeel oil and 5 % soybean oil when compared with a comparator groups fed a diets with 12 % soybean oil^(34)^.

Male C57BL/6J mice fed a high fat diet containing 15 % pollock oil and 17 % lard had lower plasma concentrations of TC and LDL-cholesterol and a lower liver TC content compared with the comparator group fed a diet containing 32 % lard^(37)^. The HDL-cholesterol concentration and the hepatic gene expression of ApoA were similar between the pollock oil group and the comparator group, whereas the hepatic mRNA levels of HMG-CoA reductase, SREBP2 and ApoB were lower in the pollock oil group^(37)^.

Saury oil was tested in three experiments in mice. Plasma TC and LDL-cholesterol concentrations were lower in male KK-A^y^ mice fed a diet with 10 % saury oil for 4 weeks compared with mice fed a diet with 10 % soybean oil^(39)^, and in male C57BL/6J mice fed a high-fat diet containing 10 % saury oil and 22 % lard for 6 weeks when compared with mice fed a diet with 32 % lard^(39)^, HDL-cholesterol was not measured in these experiments. In an experiment with a longer duration, plasma concentrations of TC, HDL-cholesterol and liver TC were not affected in male C57BL/6J mice fed a high-fat diet containing 10 % saury oil and 22 % lard at endpoint at 18 weeks when compared with the comparator group fed 32 % lard, whereas the non-HDL-cholesterol concentration was significantly lower in the saury oil group^(35)^. However, after 5 and 12 weeks intervention, the plasma TC concentration was lower in the saury oil group^(35)^.

### Effects of fish oil concentrates on markers for cholesterol metabolism

The mean plasma TC concentration was similar in Wistar rats fed a moderately high-fat diet with 6·5 % LC-MUFA concentrate and 13 % lard compared with comparator groups fed a corresponding diet with 19·5 % lard^(31,32)^. HDL-cholesterol and LDL-cholesterol were not measured in these experiments, but liver TC concentration was similar between the experimental groups^(31)^.

Male C57BL/6J mice fed high-fat diets with 12 % LC-MUFA concentrate and 20 % lard^(35)^ or 5 % MUFA concentrate and 27 % lard^(38)^ had a lower plasma TC concentration relative to the comparator groups fed diets with 32 % lard. Also, the concentrations of non-HDL-cholesterol^(35)^ and LDL-cholesterol^(38)^ were lower in these mice, whereas the HDL-cholesterol concentration^(35,38)^ and the hepatic TC content^(35)^ were similar to those of the comparator groups. The plasma TC concentration was also lower in male KK-A^y^ mice fed a regular diet with 4 % LC-MUFA concentrate and 3 % soybean oil relative to a comparator group fed a matching diet with 7 % soybean oil, whereas the liver TC concentration was similar between the groups^(36)^.

LC-MUFA rich fish oil concentrates were also tested in the genetically modified mouse models LDLr^−/−^and apoE^-/^-, using moderately high-fat diets with added 0·15 % cholesterol. In the female LDLr^−/−^mice, the plasma concentrations of TC, HDL-cholesterol and LDL-cholesterol and the liver TC content were not affected after consumption of diets containing 2 % LC-MUFA concentrate and 19 % milk fat compared with a diet with 21 % milk fat^(41)^, or after consumption of diets with 5·1 % CA-concentrate and 15·9 % milk fat relative to the comparator diet with 21 % milk fat^(42)^. The plasma TC and HDL-cholesterol concentrations were not different between male apoE^-/^- mice fed diets with 5·2 % LC-MUFA-concentrate plus 14·8 % milk fat and the comparator group fed a diet with 20 % milk fat; however, the LDL-cholesterol concentration was lower in the LC-MUFA group^(41)^. The hepatic CYP7A1 mRNA level was higher in both LDLr^−/−^mice and apoE^-/^ mice fed a LC-MUFA diet^(41)^.

### Effects of fish oils and fish oil concentrates on the fatty acid composition in liver

As an indication of the bioavailability of CA, we summarise the reports on CA levels in the liver after dietary intake in Table 1. We chose to focus on the liver since the fatty acid composition in plasma/serum is strongly affected by the recent feed intake (dietary fatty acids transported as triacylglycerols in chylomicrons) and the prandial status (release of NEFA from white adipose tissue to the circulation in the fasting state). CA cannot be synthesised to any significant degree in rodents; however, dietary CA can be oxidised to gadoleic acid (GA, C20:1*n*-11) in peroxisomes^(22)^. Similarly, the EPA and DHA amounts found in rodent liver largely originate from dietary intake, but can be synthesised to a limited degree from ALA (*α*-linolenic acid, C18:3*n*-3). Fatty acids were quantified in total liver lipids or liver phospholipids from eleven experiments. Diets containing saury oil^(35,39)^ or pollock oil^(37)^ resulted in higher CA^(35,37)^, GA^(35)^ or ΣC22:1^(39)^ levels in total liver lipids levels relative to their respective comparator groups. In rodents fed fish oil concentrates, CA^(35,36,38,41,42)^ and GA^(35,36,41,42)^ were higher in total liver lipids, whereas the CA level in liver phospholipids was similar to that of the comparator group^(31)^. Data on the amounts of CA and GA in liver were not available for rats fed redfish oil^(40)^, herring oil^(33)^ or sandeel oil^(34)^.

The tested CA-rich fish oils also contained *n*-3 LC-PUFA, and this was reflected in higher hepatic levels of EPA^(34,37,39)^, DHA^(34,37,39)^ and of Σ*n*-3 PUFA^(35)^. The LC-MUFA concentrates contained no or very low amounts of *n*-3 LC-PUFA, but still, higher EPA^(38,41)^ and DHA^(31,35,38,41)^ levels were detected relative to their comparator groups in some studies, whereas others detected no differences between LC-MUFA groups and their comparator groups for levels of EPA^(31,32,35,36,42)^ and DHA^(32,42)^, or a lower DHA level^(36)^.

### Dietary intake and adiposity

The inclusion of fish oil or fish oil concentrate in the diets may affect dietary intake, and also the high contents of lard and milk fat in some of the diets may affect the amount of feed consumed by the rodents, and may in turn influence biochemical parameters and physiological outcomes including adiposity. Six articles reported that the feed or energy intake was similar between the intervention and comparator groups^(31,35–38,40)^, in one article the intake was measured but statistical testing was not performed^(32)^ and five articles gave no information the dietary intake^(33,34,39,41,42)^.

Adiposity was measured in five articles, comprising in total six experiments^(34,36–39)^ (online Supplemental Table 1). Relative to the comparison groups, the mesenteric fat mass was lower in KK-A^y^ mice fed saury oil^(39)^, and the relative weights of epididymal and subcutaneous WAT were lower in KK-A^y^ mice fed LC-MUFA concentrate with no difference from comparator group for the relative mesenteric WAT weight^(36)^. The absolute or relative weights of adipose tissues were similar in C57BL/6J mice fed pollock oil^(37)^, in C57BL/6J mice fed saury oil^(39)^, in Zucker fa/fa rats fed sandeel oil^(34)^ and in C57BL/6J mice fed LCMUFA concentrate^(38)^ when compared to their respective comparator group.

### Meta-analyses

**Fig. 2.**
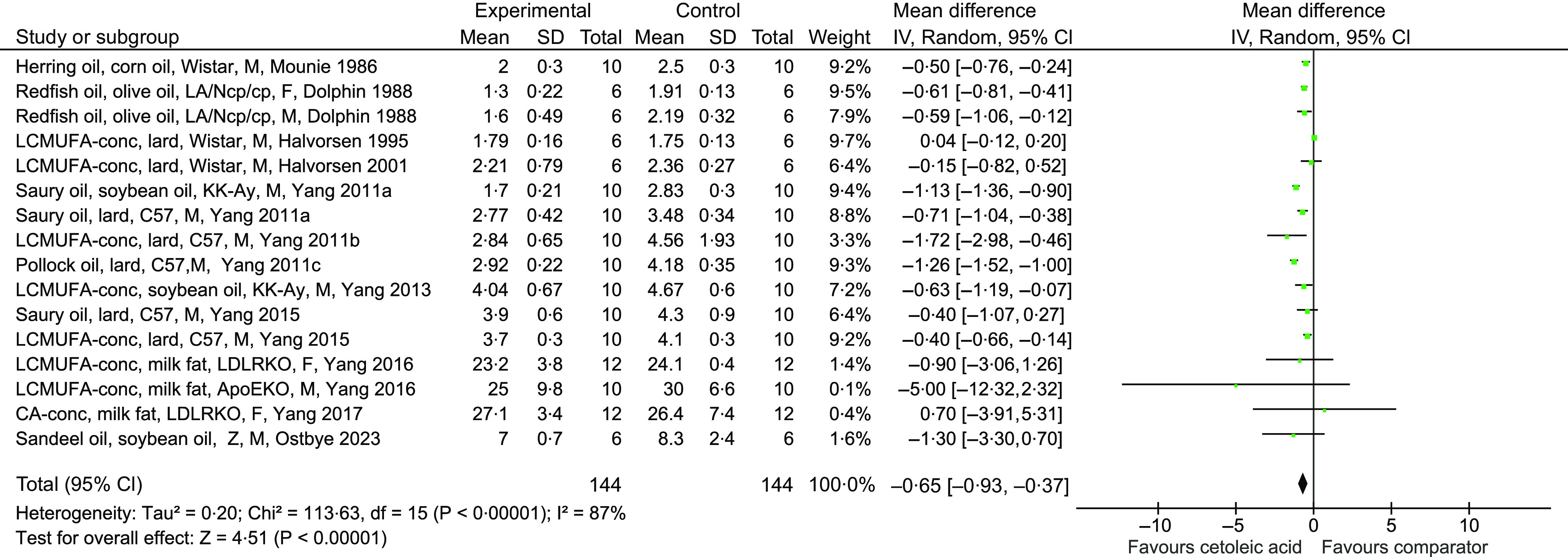
Meta-analysis using a random effects model presenting the effects of intake of CA-rich fish oils and concentrates on circulating total cholesterol concentration (mmol/l) as a forest plot. CI; confidence interval. The studies are described as intervention diet (type of fish oil or concentrate), comparator diet (type of fat), rodent strain, sex of the rodents, first author and year of publication. Abbreviations used: Type of fish or concentrate: LCMUFA-conc; long-chain monounsaturated fatty acid concentrate, CA-conc; cetoleic acid concentrate. Sex of the rodents: F; female, M; male. Rodent strain: apoE; apolipoprotein E null mice, C57; C57BL/6J mice, KKAy; KK-A^y^ mice, LDLRKO; LDL receptor knockout mice, LE; W; Wistar rats, Z; Zucker fa/fa rats.

The main meta-analysis of differences in endpoint serum/plasma TC concentration in the intervention and comparator groups includes data from all twelve reviewed articles, comprising in total sixteen experiments and representing findings in 144 rodents in the intervention groups and 144 rodents in the comparator groups (Fig. 2). The meta-analysis revealed that consumption of diets containing CA-rich fish oils or fish oil concentrates resulted in lower circulating TC concentration in rodents compared with their respective comparators, with a mean difference −0·65 mmol/l and 95 % confidence interval −0·93, −0·37 mmol/l, with an overall test for effect *Z* = 4·51 and *P* < 0·00001. When testing for statistical heterogeneity, *χ* ^2^ was 113·63 (*P* < 0·00001) and *I* ^2^ was 87 %, thus reflecting that both the direction of the between-group differences and the magnitude of effect were highly heterogeneous in our meta-analysis.

**Fig. 3.**
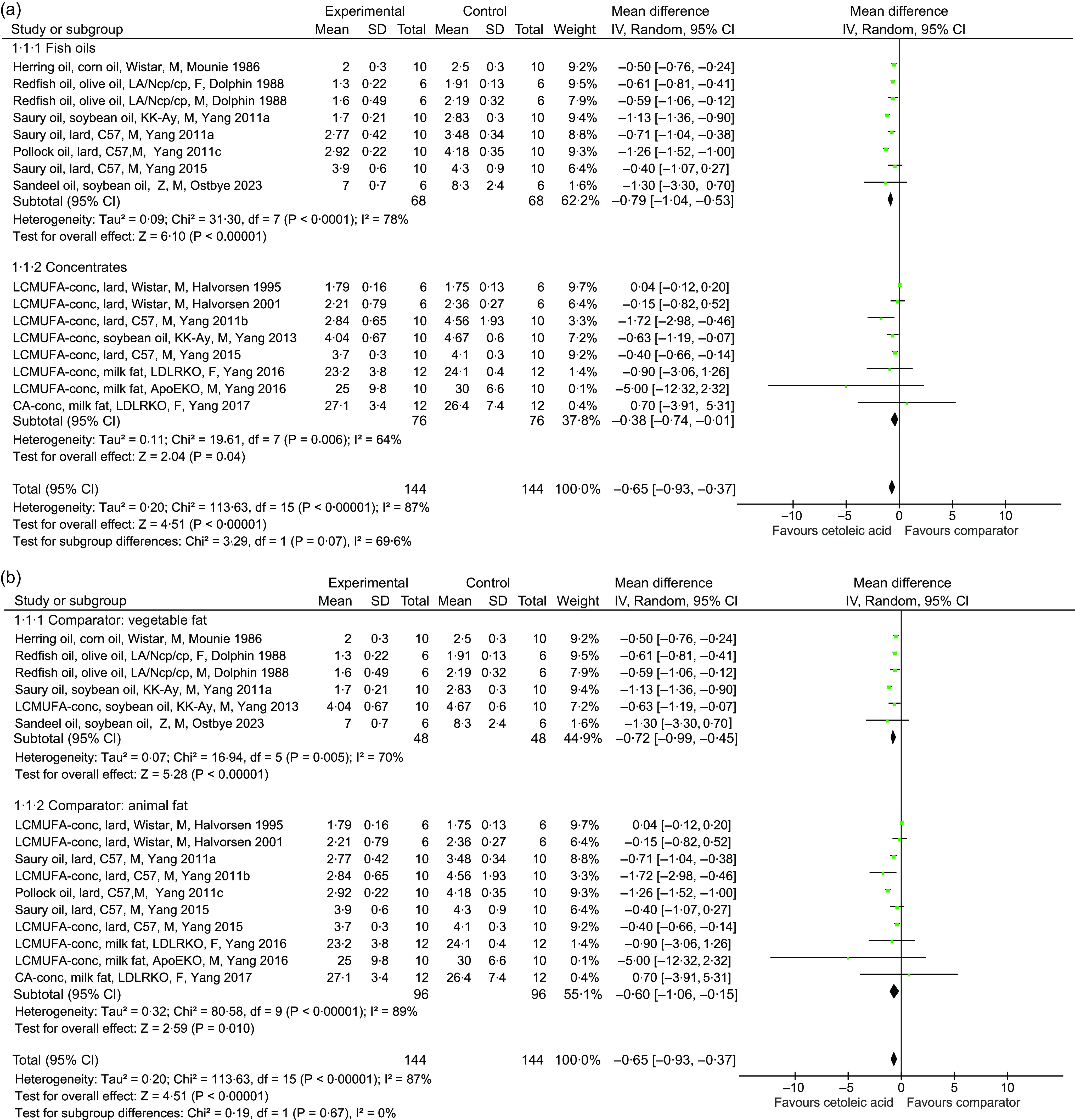
Subgroup analyses for meta-analysis using a random effects model presenting the effects of intake of CA-rich fish oils and concentrates on circulating total cholesterol concentration (mmol/l) as a forest plot; experiments using intervention diets containing fish oil *v*. diets containing LC-MUFA or CA concentrates (a), experiments using comparison diets containing vegetable fat (corn oil, olive oil, or soybean oil) *v* diets containing animal fat (lard or milk fat) (b). CI; confidence interval. The studies are described as intervention diet (type of fish oil or concentrate), comparator diet (type of fat), rodent strain, sex of the rodents, first author and year of publication. Abbreviations used: Type of fish or concentrate: LCMUFA-conc; long-chain monounsaturated fatty acid concentrate, CA-conc; cetoleic acid concentrate. Rodent strain: apoE; apolipoprotein E null mice, C57; C57BL/6J mice, KKAy; KK-A^y^ mice, LDLRKO; LDL receptor knockout mice, LE; W; Wistar rats, Z; Zucker fa/fa rats. Sex of the rodents: F; female, M; male.

The number of included experiments in the present meta-analyses was relatively low, with sixteen experiments in total, therefore only two subgroup analyses were conducted to explore the heterogeneity in the main meta-analysis. First, the effects of diets containing fish oil and diets containing LC-MUFA or CA concentrates on serum/plasma TC concentration were tested separately and gave a mean difference (95 % CI) of −0·79 (−1·04, −0·53) mmol/l for fish oils and −0·38 (−0·74, −0·01) mmol/l for concentrates, with *P* < 0·0001 (*Z* = 6·10) and *P* 0·04 (*Z* = 2·04), respectively, relative to the comparator groups. The heterogeneity was still high for both subgroups, with *χ* ^2^ = 31·30 (*P* < 0·0001) and *I* ^2^ = 78 % for fish oils and *χ* ^2^ = 19·61 (*P* 0·006) and *I* ^2^ = 64 % for concentrates. When the subgroups were compared, no difference was detected between the effect of concentrates and fish oils on circulating TC concentration (*P* 0·07, Fig. 3(a)).

Next, a subgroup analysis was conducted to investigate whether the fat sources in the comparison groups affected the outcome of the intervention studies, by comparing the intervention groups to comparator groups fed diets containing either vegetable fat (corn oil, olive oil or soybean oil) or animal fat (lard or milk fat). The comparisons to both vegetable fat and animal fats resulted in significantly lower serum/plasma TC concentration in rodents fed fish oils or concentrates relative to comparator diets containing vegetable fat (mean difference −0·72 (−0·99, −0·45) with *P* < 0·00001) or animal fat (mean difference −0·60 (−1·06, −0·15) with *P* 0·010), with no significant difference between the subgroups (*P* 0·67, Fig. 3(b)). Both analyses revealed that the heterogeneity was high for both subgroup comparisons, with *χ* ^2^ = 16·94 (*P* < 0·005) and *I* ^2^ = 70 % for comparator diets containing vegetable fat and *χ* ^2^ = 80·58 (*P* < 0·00001) and *I* ^2^ = 89 % for comparator diets containing animal fat.

The sensitivity analysis, conducted through a leave-one-out analysis of studies with the highest positive and negative effect sizes, and the studies with the highest risk of bias or lowest quality of evidence were excluded sequentially from the meta-analysis, did not alter the outcome measure (data not presented).

**Fig. 4.**
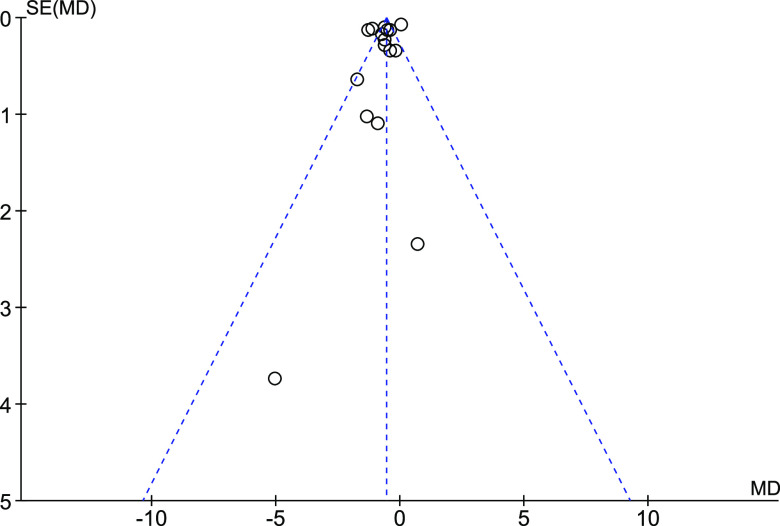
Funnel plot showing the effect estimate with 95 % CI for the effect of intake of diets containing CA-rich fish oils or concentrates on circulating total cholesterol concentration.

The asymmetry of the funnel plot (Fig. 4) was visually inspected to assess the possibility that results are missing from our meta-analysis, and the based on the low asymmetry, we consider that the risk for publication bias is low.

## Discussion

In this systematic review with meta-analysis, we present evidence for a beneficial effect of CA on the cholesterol metabolism in rodents, showing that there is more to fish oils than EPA and DHA. The present review is based on twelve articles, comprising in total sixteen experiments and 288 rodents, with variations in three of the components described in our PICO strategy; i.e. a variety of rat and mouse strains and genetically modified rodents were used, a variety of fish oils and fish oil concentrates with different CA content were explored in the interventions, and the comparator diets contained different types of vegetable or animal fats. All the included articles reported the effects of the intervention on the concentration of TC in serum or plasma. To the best of our knowledge, this is the first systematic review and meta-analysis that explores the reported effects of dietary CA on the circulating TC concentration in rodents.

The meta-analysis showed that rodents fed a diet containing CA had a significantly lower circulating TC concentration relative to their respective comparator group, with an average lower total TC concentration of −0·65 mmol/l. This corresponds to a 16 % lower TC concentration in the CA-fed rodents, and although not directly transferable to humans, this may be relevant and should be further explored in humans with a high risk for hypercholesterolaemia since a TC reduction of 3 % is sufficient to induce a reduction in CHD risk by 15 %^(49)^.

The heterogeneity in the meta-analysis was large, as could be expected from the variations in designs of the included studies, despite our effort to minimise the heterogeneity in design by applying a strict list of exclusion and inclusion criteria. Although the use of funnel plot is not recommended when the statistical heterogeneity is large (>50 %)^(50)^, as is the case for the present meta-analysis with *I* ^2^ of 89 %, the low asymmetry in the funnel plot suggest that the risk for publication bias was low.

The main meta-analysis contained sixteen feeding experiments; therefore, we chose to conduct only two subclass analyses. The similar effect of fish oils and fish oil concentrates on circulating TC concentration is of interest since the CA content in the diets were in the range 0·72–1·97 g/100 g diet in the fish oil diets, and 0·70–4·78 g/100 g diet in diets containing concentrates of CA or LC-MUFA, whereas the EPA + DHA amount was in the range 0·67–2·74 in the fish oil diets and negligible in the diets that were added concentrates. This indicates that CA and not *n*-3 PUFAs were responsible for the lower circulating TC concentration in the main meta-analysis.

In some of the included experiments, the effect of fish oil or LC-MUFA concentrates on circulating TC concentration was compared with that of a recognised unhealthy type of animal fat, whereas others tested against more neutral oils of vegetable origin. In the present meta-analyses, we have included comparison groups containing vegetable fat (corn oil, olive oil or soybean oil) or animal fat (lard or milk fat). Both lard and milk fat contain cholesterol in addition to saturated fatty acids and can be expected to rise TC concentration in rodents, however, also fish oils contain cholesterol and saturated fatty acids^(51)^. The subgroup meta-analyses showed no difference between intervention diets relative to the comparison diets containing vegetable fat or those containing animal fat. The finding that diets containing CA beneficially affects circulating TC concentration when compared with both vegetable and animal fats strengthens our findings and makes the result more generalisable.

The circulating TC concentration is regulated by changes in the endogenous production of cholesterol in the liver, the uptake of LDL-cholesterol to the liver, the amount stored as cholesteryl esters in the liver, the secretion of VLDL from the liver and the excretion of cholesterol and bile acids in faeces, and these pathways are affected by dietary intakes of cholesterol, fats and other nutrients. We have recently shown that diets containing fish proteins prevent high cholesterol concentration in rodents, most likely by increased faecal removal of TC and/or bile acids in normocholesterolemic rodents and through down-regulation of hepatic cholesterol synthesis hypercholesterolaemic Zucker fa/fa rats^(52)^. It was therefore mandatory that the included articles in the present review had similar amount and type of protein in the intervention diet and the comparator diet. Cholesterol intake affect circulating TC concentration in C57BL/6 mice at already a low dietary dose of 0·1 % cholesterol through suppression of HMG-CoA reductase activity and a reduction in the fraction of cholesterol absorbed, and by increasing CYP7A1 activity followed by higher faecal bile acid excretion^(53)^. The cholesterol content was not measured in any of the intervention and comparator diets included in the present systematic review, and the cholesterol content was not balanced between intervention diet and comparator diets in any of the experiments. An estimation of the cholesterol content in the diets, based on available data cholesterol contents in fats and oils^(51)^, suggests that the cholesterol content was in the range 0·01–0·20 mg/100 g diet for all diets with little differences between intervention and comparator diets, which may be too low to influence the cholesterol metabolism.

The primary target for medical treatment of hypercholesterolaemia is to reduce the endogenous synthesis of cholesterol in the liver by inhibiting the activity of HMG-CoA reductase^(54)^. A lower cholesterol biosynthesis will reduce the amount of cholesterol that can be secreted from liver in VLDL particles and stimulate the hepatic LDL receptor. The activity or gene expression of LDL receptor was not investigated in any of the included articles. The effect on HMG-CoA reductase was investigated in one article, showing lower gene expressions of both of HMG-CoA reductase and of SREBP2, which regulates transcription of the HMG-CoA reductase gene, in the liver of C57BL/6J mice fed pollock oil^(37)^. Concomitant with this, the hepatic ApoB mRNA level was lower in the pollock oil fed mice, suggesting a lower capacity for producing VLDL, and this may explain the lower plasma concentrations of TC and LDL-cholesterol as well as the lower TC content in liver from in these mice^(37)^ and the lower VLDL concentration in both male and female LA/N *cp/cp* rats fed redfish oil^(40)^.

The endogenous cholesterol synthesis is also strongly correlated with adiposity^(55)^, and a decrease in the amount of adipose tissue will efficiently lower the circulating TC concentration in humans with overweight or obesity^(56)^. The adiposity of the rodents was measured in five articles, which reported of no or only partial effect of consumption of CA-rich fish oils or concentrates^(34,36–39)^, suggesting that the lower TC concentration was not regulated through down-regulation of HMG-CoA reductase. Due to the limited data on the effects of consuming CA-rich fish oils and concentrates on HMG-CoA reductase, the role of endogenous cholesterol synthesis on the regulation of the circulating concentration of TC is difficult to assess.

The dietary intake of cholesterol contributes to less than half of the cholesterol available for intestinal absorption in adult humans, whereas 800 to 1200 mg cholesterol/d origins from the biliary tract^(57)^. In rats, it was demonstrated that the intestinal cholesterol absorption was lower when diets contained the LC-MUFA erucic acid (C22:1*n*-9) compared with diets containing lard, and was inversely correlated with faecal sterol excretion^(58)^. Thus, the lower TC concentration in the intervention groups in the meta-analyses may have been caused by the presence of CA in the diets through increasing the faecal excretion of cholesterol, rendering less cholesterol available for secretion as VLDL or storage as cholesteryl esters in the liver. Although none of the included studies measured the faecal excretion of cholesterol or bile acids or SOAT2, the higher hepatic mRNA level of CYP7A1 in LDLr^-/^- mice and apoE^-/^ mice fed LC-MUFA concentrate^(41)^ suggests that the biosynthesis of bile acids from cholesterol in the liver, and consequently the faecal bile acid excretion, was increased relative to the comparator group and may contribute to a lower circulating TC concentration. Others have shown that the lower TC concentration in LDLr^−/−^mice after intake of herring fillet was accompanied with a higher hepatic gene expression of CYPA7A when compared with a control group feed beef^(20)^. Thus, since faecal excretion of neutral and acidic steroids is the major route of cholesterol removal from the body, an upregulation of this pathway may be one explanation for the lower TC concentration after CA intake.

Vertebrates have limited capacity to biosynthesise CA; therefore the amount of CA in the liver will be indicative of uptake of CA from the diet. Quantification of CA in liver was conducted in eight articles, showing higher amounts of CA^(35–38,41,42)^ or ΣC22:1^(39)^ in total liver lipids compared with their respective comparator group, whereas the CA level in liver phospholipids was similar to that of the comparator group in one article^(31)^. Gadoleic acid, a 20 carbon long oxidation product of CA^(22)^, was also found in liver of rodents fed CA-rich diets including when no gadoleic acid was present in the intervention diet^(42)^, further supporting an intestinal absorption of CA from diets containing fish oil or fish oil concentrate.

Fish oils are most known for the contents of the n-3 PUFAs, especially EPA and DHA. As could be expected, rodents consuming diets containing saury oil^(35,39)^, pollock oil^(37)^ or sandeel oil^(34)^, which all contain EPA and DHA, had higher levels of these fatty acids in liver. More surprising were the higher hepatic EPA^(38,41)^ and DHA^(31,35,38,41)^ levels in rodents fed LC-MUFA concentrates containing low amounts of EPA and DHA, although others detected no differences between LC-MUFA groups for levels of EPA^(31,32,35,36,42)^ and DHA^(32,42)^, or even a lower DHA level^(36)^. Findings in cell culture studies suggest that CA may stimulate the endogenous synthesis of EPA and DHA from ALA by upregulating the delta-5 and delta-6 desaturases (fatty acid desaturase 1 and 2, respectively) in the liver^(59)^. An upregulation of desaturases is a plausible explanation as all of these diets contained ALA, although the experiments demonstrating higher EPA and/or DHA levels in liver used diets containing some EPA and DHA. However, the only experiment using an EPA- and DHA-free CA-concentrate did not observe a higher hepatic levels of EPA and DHA^(42)^. EPA and DHA downregulate the activities of delta-5 and delta-6 desaturases through feedback inhibition at both gene expression and enzyme activity levels^(60)^ and this may explain the lower gene expressions of these desaturase enzymes measured in livers from rats fed sandeel oil^(34)^.

More research on possible health effects of fish oil and fish oil concentrates containing CA is warranted. Experiments conducted before the mid-1990s may have given CA an undeserved bad reputation, by testing partially hydrogenated fish oil (often from herring) with a high content of CA but also containing harmful trans-fatty acids such as cetelaidic acid (trans-C22:1*n*-11) and brassidic acid (trans-C22:1*n*-9). As reviewed by Bremer and Norum i 1982, consumption of hydrogenated fish oils induces accumulation of triacylglycerol in extrahepatic tissues, especially in the myocardium^(61)^. To the best our knowledge, pure CA or concentrates of CA or LC-MUFA has not been tested in humans, but saury oil was tested in two experiments in healthy adults, using a daily dose of 4·2 g^(62)^ or 12 g^(63)^ of saury oil. In both experiments, the intervention supplement and the comparator supplement were matched with similar contents of *n*-3 LC-PUFA from either tuna oil^(62)^ or sardine oil^(63)^ with the intention to isolate the effect of the LC-MUFAs in the saury oil. However, neither study detected differences between the experimental groups with regard to TC, HDL-cholesterol and LDL-cholesterol, but concluded that a LC-MUFA supplement is safe and show promising effects on the lipoprotein profile in healthy humans^(62,63)^.

Unlike humans, mice and rats are naturally deficient in cholesteryl ester transfer protein, and whereas LDL particles are the major cholesterol transporter in humans^(64)^, rodents use HDL as the major transporter for cholesterol^(65)^. In many other aspects, the cholesterol concentration in circulation, liver and extrahepatic tissues is controlled through similar pathways in rodents and humans, including by regulation of the activities of HMG-CoA reductase and LDL receptor, and through faecal excretion of cholesterol and bile acids, with the liver as the primary site for endogenous cholesterol production and faecal excretion of bile acids as the major route of cholesterol removal from the body. High energy diets and diets with added extra cholesterol fed to rodents are of relevance as they bear similarities to the Western diet with high content of fats, carbohydrates and cholesterol^(66,67)^. Also, the possibility of using genetically modified mice such as the apoE-/- mouse^(46)^ and the LDLr^-/^- mouse^(47)^ are relevant models of hypercholesterolaemia since LDL removal is inhibited and thus the cholesterol metabolism is disturbed and share similarities with spontaneous atherosclerosis^(46)^ and familial hypercholesterolaemia^(47)^ seen in humans. Although dietary studies in rodents are relevant for humans, the findings in rodents must be interpreted with caution regarding the relevance to human physiology, as there are substantial differences between rodents and humans that limit the reliability and translation of findings from rodents to humans.

The risk of bias for the included studies is difficult to assess, as the majority of the entries in the SYRCLE’s risk of bias tool^(25)^ were not addressed in the articles and therefore were graded as having an unclear risk of bias to the reported findings. We did not detect lack of reporting outcome data, which strengthens the findings. The absence of information regarding blinding is a cause for concern, as absence of blinding could influence the interpretation of results and since non-blinded studies have been found to produce more statistically significant findings^(68)^. Still, we considered the included studies is to be of high quality with regard to the primary aim of the present systematic review and meta-analysis, based on the high average score using the selected items from the CAMARADES checklist^(27)^ and the ARRIVE 2·0 guidelines^(28)^.

### Strengths and limitations

The finding that rodents fed diets containing CA-rich fish oils or concentrates had lower circulating concentration of TC is strengthened by the subgroup analysis showing that this is statistically significant relative to both comparator diets containing vegetable fat (corn oil, olive oil or soybean oil) or animal fat (lard or milk fat). The exclusion of articles using comparator diets that differed from the intervention diet with regard to type and/or amount of proteins and carbohydrates further eliminates effects caused by ingredients other than the CA-rich fish oils or concentrates. This is further supported by the subgroup comparison showing no difference between CA-rich fish oil and CA-rich fish oil concentrate, as the content of *n*-3 LC-PUFA was higher in the former group. The CA doses used in the included intervention diets are relatively low; from 0·70 to 4·78 g CA per 100 g diet, corresponding to 1·3–9·5 % of energy. Also the energy contribution from EPA + DHA in the included articles was low, from 0·02 to 4·88 % of energy in the diets. Thus, the contribution from CA and EPA + DHA to the dietary energy intake is markedly lower compared with the necessary dose of 10–40 % of energy from EPA and DHA that is required to lower the cholesterol concentration in rodents^(17)^.

Baseline TC concentrations were reported in only two of the reviewed articles^(35,37)^; however, no statistical comparisons between baseline and endpoint values were conducted. Although we can assume that the TC concentration increased during the intervention period based on a predisposition for developing hypercholesterolaemia in certain rodent strains and the use of hypercholesterolaemic diets in some of the included studies, we cannot conclude whether CA intake prevents or attenuates an increase in TC concentration, or if CA lowers the TC concentration.

There are some limitations to the interpretation of the present systematic review and the meta-analysis that should be considered; such as the limited number of articles, the variety in CA-doses and rodent models that were used, and the diversity of the duration of the interventions. Animal fats, including fish oils, lard and milk fat typically contain some cholesterol; however, none of the included experiments balanced the cholesterol contents in the intervention diet with the comparator diet to ensure similar cholesterol intake between the experimental groups. In addition, of the 288 experimental rodents used in the included articles, only sixty rodents (21 %) were females, which make the results from the present study less generalisable.

### Conclusion

Based on the meta-analysis, we conclude that dietary intake of CA-rich fish oils or concentrates results in lower circulating TC concentration in rodents. The subgroup analyses indicate that this effect is independent of *n*-3 LC-PUFA content in the CA-rich fish oils and concentrates and also applies independently of whether the comparator fat is of animal origin (considered cholesterologenic) or from plants. The potential of CA to prevent high TC concentration in rodents makes this an interesting fatty acid for future research in humans with high risk for developing CVD. We consider the publication bias to be low for the meta-analysis and the quality of the included studies to be high but with an uncertain risk of bias.

## Supplementary material

For supplementary material accompanying this paper visit https://doi.org/10.1017/S0007114523002118. click here to view supplementary material
