# Supplementary material for: Effects of diets containing fish oils or fish oil concentrates with high cetoleic acid content on the circulating cholesterol concentration in rodents. A systematic review and meta-analysis
Source: Br J Nutr. 2023 Sep 22;131(4):606–21. doi: 10.1017/S0007114523002118 (PMC10803824; doi:10.1017/S0007114523002118)
Supplement: Supplementary file 1 [file S0007114523002118sup.zip › S0007114523002118sup001.docx]

**Supplemental Table 1:** Study characteristics; type fish used for preparation of oils and concentrates), length of intervention period, diet availability, prandial state at blood sampling and outcomes (dietary intake, adiposity)

| Ref. | Fish species (w. Latin name when provided) or genus, part of fish used and processing, name of supplier | Duration of intervention period | Diet availability | Prandial state at blood sampling (euthanasia or at retro-orbital blood sampling) | Dietary intake in intervention group relative to comparator group | Adiposity in intervention group relative to comparator group |
| --- | --- | --- | --- | --- | --- | --- |
| Mounie J.  1986  ^(^[^33^](#_ENREF_33)^)^ | Fish oil from North Sea herring | 40 days | Ad libitum | Fasted 16 hrs | N/A | N/A |
| Dolphin P.J.  1988  ^(^[^40^](#_ENREF_40)^)^ | Redfish (Sebastes marinus) oil derived from the viscera and non-saleable portions (Vitashine Ltd., Nova Scotia) | Both experiments:  8 weeks | Both experiments:  Ad libitum | Both experiments:  Fasted overnight | Both experiments:  Energy intake: NS | Both experiments:  N/A |
| Halvorsen B. 1995  ^(^[^31^](#_ENREF_31)^)^ | Fish oil concentrate of ethyl esters of very long-chain mono- unsaturated fatty acids (Pronova, Oslo, Norway) | 3 weeks | Ad libitum | Non-fasted | Feed intake: NS | N/A |
| Halvorsen B. 2001  ^(^[^32^](#_ENREF_32)^)^ | Fish oil concentrate of ethyl esters of very long-chain mono- unsaturated fatty acids (Pronova, Oslo, Norway) | 3 weeks | Ad libitum | Non-fasted | Not tested statistically | N/A |
| Yang Z.H.  2011  ^(^[^37^](#_ENREF_37)^)^ | Pollock oil (Nippon Suisan Kaisha, Ltd., Tokyo, Japan) | 6 weeks. | N/A | N/A | Feed intake: NS | Epididymal and mesenteric relative WAT weights: NS |
| Yang Z.H.  2011  ^(^[^38^](#_ENREF_38)^)^ | Saury oil concentrate of MUFA ethyl esters (Nippon Suisan Kaisha, Ltd., Tokyo, Japan) | 6 weeks | Ad libitum | Non-fasted | Feed intake: NS | Mesenteric fat mass: NS |
| Yang Z.H.  2011  ^(^[^39^](#_ENREF_39)^)^ | Saury oil (Nippon Suisan Kaisya, Ltd., Tokyo, Japan) | Exp.1:  4 weeks  Exp. 2:  6 weeks | Both experiments:  pair-feeding | Both experiments:  Non-fasted | Both experiments:  N/A | Exp. 1:  Mesenteric fat mass: lower in saury oil group  Exp. 2:  Mesenteric fat mass: NS |
| Yang Z.H.  2013  ^(^[^36^](#_ENREF_36)^)^ | Saury oil concentrate of LC-MUFAs (Nippon Suisan Kaisha, Ltd. (Tokyo, Japan) | 8 weeks | N/A | Non-fasted | Feed intake: NS | Epididymal and subcutaneous relative WAT weights: lower in LC-MUFA group  Mesenteric relative WAT weight: NS |
| Yang Z.H.  2015  ^(^[^35^](#_ENREF_35)^)^ | Saury oil and fractions of fish oil concentrate of LC-MUFA ethyl esters (Nippon Suisan Kaisha, Ltd., Tokyo, Japan). | Exp. 1:  18 weeks  Exp. 2:  8 weeks | Both experiments:  Ad libitum | Both experiments:  Fasted 5 hrs | Both experiments:  Feed intake: NS | Both experiments:  N/A |
| Yang Z.H.  2016  ^(^[^41^](#_ENREF_41)^)^ | LC-MUFA concentrate was supplied by Nippon Suisan Kaisha (Tokyo, Japan) | Both experiments:  12 weeks | Both experiments:  N/A | Both experiments:  Fasted 5 hrs | Both experiments:  N/A | Both experiments:  N/A |
| Yang Z.H.  2017  ^(^[^42^](#_ENREF_42)^)^ | Saury oil concentrate of CA ethyl ester (Nippon Suisan Kaisha, Tokyo, Japan) | 12-week | N/A | Non-fasted | N/A | N/A |
| Østbye T.K. 2023  ^(^[^34^](#_ENREF_34)^)^ | Sandeel oil (Vedde AS, Langevåg, Norway) | 4 weeks | Ad libitum | Non-fasted | N/A | Epididymal WAT weight: NS |

NS; not statistically significant, N/A, data not available, WAT, white adipose tissue
