# Supplementary material for: Effects of diets containing fish oils or fish oil concentrates with high cetoleic acid content on the circulating cholesterol concentration in rodents. A systematic review and meta-analysis
Source: Br J Nutr. 2023 Sep 22;131(4):606–21. doi: 10.1017/S0007114523002118 (PMC10803824; doi:10.1017/S0007114523002118)
Supplement: Supplementary file 1 [file S0007114523002118sup.zip › S0007114523002118sup002.docx]

**Supplemental table 2:** Evaluation of the risk of bias for the included articles (Yes; low risk of bias, No; high risk of bias, Unclear; Unclear risk of bias)

|  | SYRCLES items with signalling questions | | | | | | | | | |
| --- | --- | --- | --- | --- | --- | --- | --- | --- | --- | --- |
|  | 1. Sequence generation (selection bias) | 2. Baseline characteristics (selection bias) | 3. Allocation concealment (selection bias) | 4. Random housing (performance bias) | 5. Blinding (performance bias) | 6. Random outcome assessment (detection bias) | 7. Blinding (detection bias) | 8. Incomplete outcome data (attrition bias)* | 9. Selective outcome reporting (reporting bias) | 10. Other sources of bias |
|  | Was the allocation sequence adequately generated and applied? | Were the groups similar at baseline, or were they adjusted for confounders in the analysis? | Was the allocation adequately concealed? | Were the animals randomly housed during the experiment? | Were the caregivers and/or investigators blinded from knowledge which intervention each animal received during the experiment? | Were animals selected at random for outcome assessment? | Was the outcome assessor blinded? | Were incomplete outcome data adequately addressed? | Are reports of the study free of selective outcome reporting? | Was the study apparently free of other problems that could result in a high risk of bias? |
| **^(^**[**^33^**](#_ENREF_33)**^)^** | Unclear | Unclear | Unclear | Yes | Unclear | Unclear | Unclear | Yes | Unclear | Unclear |
| **^(^**[**^40^**](#_ENREF_40)**^)^** | Unclear | Unclear | Unclear | Yes | Unclear | Unclear | Unclear | Yes | Unclear | Unclear |
| **^(^**[**^31^**](#_ENREF_31)**^)^** | Unclear | Unclear | Unclear | Yes | Unclear | Unclear | Unclear | Yes | Unclear | Unclear |
| **^(^**[**^32^**](#_ENREF_32)**^)^** | Unclear | Unclear | Unclear | Yes | Unclear | Unclear | Unclear | Yes | Unclear | Unclear |
| **^(^**[**^37^**](#_ENREF_37)**^)^** | Unclear | Yes | Unclear | Yes | Unclear | Unclear | Unclear | Yes | Unclear | Unclear |
| **^(^**[**^38^**](#_ENREF_38)**^)^** | Unclear | Yes | Unclear | Yes | Unclear | Unclear | Unclear | Yes | Unclear | Unclear |
| **^(^**[**^39^**](#_ENREF_39)**^)^** | Unclear | Unclear | Unclear | Yes | Unclear | Unclear | Unclear | Yes | Unclear | Unclear |
| **^(^**[**^36^**](#_ENREF_36)**^)^** | Unclear | Yes | Unclear | Yes | Unclear | Unclear | Unclear | Yes | Unclear | Unclear |
| **^(^**[**^35^**](#_ENREF_35)**^)^** | Unclear | Yes | Unclear | Yes | Unclear | Unclear | Unclear | Yes | Unclear | Unclear |
| **^(^**[**^41^**](#_ENREF_41)**^)^** | Unclear | Unclear | Unclear | Yes | Unclear | Unclear | Unclear | Yes | Unclear | Unclear |
| **^(^**[**^42^**](#_ENREF_42)**^)^** | Unclear | Unclear | Unclear | Yes | Unclear | Unclear | Unclear | Yes | Unclear | Unclear |
| ^(^[^34^](#_ENREF_34)^)^ | Unclear | Yes | Unclear | Yes | Unclear | Unclear | Unclear | Yes | Unclear | Unclear |

*For main outcome in the present systematic review and meta-analysis, i.e., serum/plasma total cholesterol concentration
