# Supplementary material for: Effects of diets containing fish oils or fish oil concentrates with high cetoleic acid content on the circulating cholesterol concentration in rodents. A systematic review and meta-analysis
Source: Br J Nutr. 2023 Sep 22;131(4):606–21. doi: 10.1017/S0007114523002118 (PMC10803824; doi:10.1017/S0007114523002118)
Supplement: Supplementary file 1 [file S0007114523002118sup.zip › S0007114523002118sup003.docx]

**Supplemental Table 3:** Study quality checklist for the included studies. Studies were scored with maximum one point per item when the information was provided and with zero points if the information was missing.

| Refs. | 1. Peer reviewed publication | 2. Animal model (specie, strain) | 3. Sex of the experimental animals | 4. Housing and husbandry conditions (animals per cage, temperature and light cycle), any actions to improve animal welfare of the experimental animals | 5. Description of the procedures, including when it was done, any preparation of the animals before blood sampling (prandial status, use of anaesthesia) | 6. Description of analysis or name and/or brand of kits | 7. Details of the statistical methods used for each analysis | 8. Summary/  descriptive statistics for each experimental group, with a measure of variability where applicable | 9. Compliance with animal welfare regulations | 10. Statement of potential conflict of interests | Total score (of 10) |
| --- | --- | --- | --- | --- | --- | --- | --- | --- | --- | --- | --- |
| **^(^**[**^33^**](#_ENREF_33)**^)^** | 1 | 1 | 1 | 0.5^*†^ | 0.5^\|\|^ | 1 | 1 | 1 | 0 | 0 | 7.00 |
| **^(^**[**^40^**](#_ENREF_40)**^)^** | 1 | 1 | 1 | 0.5^†‡^ | 1 | 1 | 1 | 1 | 0 | 0 | 7.50 |
| **^(^**[**^31^**](#_ENREF_31)**^)^** | 1 | 1 | 1 | 0^*†‡§^ | 1 | 1 | 1 | 1 | 0 | 0 | 7.00 |
| **^(^**[**^32^**](#_ENREF_32)**^)^** | 1 | 1 | 1 | 0.75^†^ | 0.5^\|\|^ | 1 | 1 | 1 | 0 | 0 | 7.25 |
| **^(^**[**^37^**](#_ENREF_37)**^)^** | 1 | 1 | 1 | 0.5^*†^ | 0.5^¶^ | 1 | 1 | 1 | 1 | 1 | 9.00 |
| **^(^**[**^38^**](#_ENREF_38)**^)^** | 1 | 1 | 1 | 0.75^†^ | 1 | 1 | 1 | 1 | 1 | 0 | 8.75 |
| **^(^**[**^39^**](#_ENREF_39)**^)^** | 1 | 1 | 1 | 0.75^†^ | 1 | 1 | 1 | 1 | 1 | 0 | 8.75 |
| **^(^**[**^36^**](#_ENREF_36)**^)^** | 1 | 1 | 1 | 0.75^†^ | 1 | 1 | 1 | 1 | 1 | 1 | 9.75 |
| **^(^**[**^35^**](#_ENREF_35)**^)^** | 1 | 1 | 1 | 0^*†‡§^ | 1 | 1 | 1 | 1 | 1 | 1 | 9.00 |
| **^(^**[**^41^**](#_ENREF_41)**^)^** | 1 | 1 | 1 | 0^*†‡§^ | 0.5^\|\|^ | 1 | 1 | 1 | 1 | 1 | 8.50 |
| **^(^**[**^42^**](#_ENREF_42)**^)^** | 1 | 1 | 1 | 0.25^*†‡^ | 0.5^\|\|^ | 1 | 1 | 1 | 1 | 1 | 8.75 |
| **^(^**[**^34^**](#_ENREF_34)**^)^** | 1 | 1 | 1 | 0.75^†^ | 1 | 1 | 1 | 1 | 1 | 1 | 9.75 |

^*^information on number of animals per cage was not provided (0.25 point subtracted)

^†^information on actions to improve animal welfare was not provided (0.25 point subtracted)

^‡^information on temperature was not provided (0.25 point subtracted)

^§^information on light cycle was not provided (0.25 point subtracted)

^||^information on anaesthesia was not provided (0.5 point subtracted)

^¶^information on fasting condition at blood sampling was not provided (0.5 point subtracted)
