# Supplementary material for: Effects of diets containing fish oils or fish oil concentrates with high cetoleic acid content on the circulating cholesterol concentration in rodents. A systematic review and meta-analysis
Source: Br J Nutr. 2023 Sep 22;131(4):606–21. doi: 10.1017/S0007114523002118 (PMC10803824; doi:10.1017/S0007114523002118)
Supplement: Supplementary file 1 [file S0007114523002118sup.zip › S0007114523002118sup004.docx]

**Supplemental Table 4**: Estimated contents of cetoleic acid, gadoleic acid, eicosapentaenoic acid, docosahexaenoic acid, alpha-linolenic acid and cholesterol in the intervention diets and the comparator diets, based on reported fatty acid composition in the oils or in the diets.

|  |  | g/100 g diet | | | | | mg/100 g diet |
| --- | --- | --- | --- | --- | --- | --- | --- |
| First author, year | Description of diets | CA | GA | EPA | DHA | ALA | Cholesterol^**^ |
| Mounie 1986 ^(^[^33^](#_ENREF_33)^)^ | Intervention diet (6% herring oil) ^*^ | 0.720 | 0.690 | 0.540 | 0.600 | 0.090 | 0.05 |
|  | Comparator diet (6% corn oil) ^†^ | 0 | 0.030 | 0 | 0 | 0.120 | 0 |
| Dolphin 1988 ^(^[^40^](#_ENREF_40)^)^  (both exp.) | Intervention diet (9.1% redfish oil) | 1.97 | 1.99 | 0.446 | 0.209 | N/A | 0.07 |
|  | Comparator diet (9.1% olive oil) ^‡^ | 0 | 0.028 | 0 | 0 | 0.069 | 0 |
| Halvorsen 1995 ^(^[^31^](#_ENREF_31)^)^ | Intervention diet (6.5% LC-MUFA concentrate, 13% lard and 1.5% soybean oil) | 2.69 | N/A | 0.021 | 0.042 | 0.231 | 0.01 |
|  | Comparator diet (19.5% lard and 1.5% soybean oil) | 0 | N/A | 0 | 0.126 | 0.273 | 0.02 |
| Halvorsen 2001 ^(^[^32^](#_ENREF_32)^)^ | Intervention diet (6.5% LC-MUFA concentrate, 13% lard and 1.5% soybean oil) | 2.16 | N/A | 0.063 | 0.042 | N/A | 0.01 |
|  | Comparator diet (19.5% lard and 1.5% soybean oil) | 0 | N/A | 0.021 | 0.042 | N/A | 0.02 |
| Yang 2011 ^(^[^37^](#_ENREF_37)^)^ | Intervention diet (15% pollock oil, 17% lard and 3.2% soybean oil)^4^ | 1.85 | N/A | 1.55 | 1.19 | 0.578 | 0.13 |
|  | Comparator diet (32% lard and 3.2% soybean oil) ^4^ | 0 | N/A | 0.006 | 0.010 | 0.563 | 0.03 |
| Yang 2011 ^(^[^38^](#_ENREF_38)^)^ | Intervention diet (5% MUFA concentrate, 27% lard and 3.2% soybean oil) ^4^ | 1.95 | N/A | 0.011 | 0.069 | 0.515 | 0.03 |
|  | Comparator diet (32% lard and 3.2% soybean oil) ^4^ | 0 | N/A | 0.006 | 0.010 | 0.563 | 0.03 |
| Yang 2011 ^(^[^39^](#_ENREF_39)^)^ Exp. 1 | Intervention diet (10% saury oil) | 1.85 | N/A | 0.611 | 1.18 | 0.122 | 0.08 |
|  | Comparator diet (10% soybean oil) | 0 | N/A | 0 | 0 | 0.755 | 0 |
| Yang 2011 ^(^[^39^](#_ENREF_39)^)^ Exp. 2 | Intervention diet (10% saury oil, 22% lard and 3.2% soybean oil) | 1.85 | N/A | 0.615 | 1.19 | 0.584 | 0.10 |
|  | Comparator diet (32% lard and 3.2% soybean oil) | 0 | N/A | 0.006 | 0.010 | 0.562 | 0.03 |
| Yang 2013 ^(^[^36^](#_ENREF_36)^)^ | Intervention diet (4% LC-MUFA concentrate and 3% soybean oil) | 1.39 | 0.672 | 0.008 | 0.020 | 0.236 | 0 |
|  | Comparator diet (7% soybean oil) | 0 | 0 | 0 | 0 | 0.532 | 0 |
| Yang 2015 ^(^[^35^](#_ENREF_35)^)^  Exp. 1 | Intervention diet (10% saury oil, 22% lard, 3.2% soybean oil) ^§^ | 1.10 | 0.740 | 1.11 | 1.23 | 0.593 | 0.10 |
|  | Comparator diet (32% lard and 3.2% soybean oil) ^§^ | 0 | 0 | 0 | 0 | 0.563 | 0.03 |
| Yang 2015 ^(^[^35^](#_ENREF_35)^)^  Exp. 2 | Intervention diet (12% LC-MUFA concentrate, 20% lard and 3.2% soybean oil) ^§^ | 4.18 | 2.02 | 0.024 | 0.048 | 0.479 | 0.02 |
|  | Comparator (with 32% lard and 3.2% soybean oil) ^§^ | 0 | 0 | 0 | 0 | 0.563 | 0.03 |
| Yang 2016 ^(^[^41^](#_ENREF_41)^)^  Exp. 1 | Intervention diet (2% LC-MUFA concentrate and 19% milk fat) ^\|\| 6^ | 0.696 | 0.355 | 0.004 | 0.008 | 0.066 | 0.19 |
|  | Comparator diet (21% milk fat) ^\|\| 6^ | 0 | 00.21 | 0 | 0 | 0.066 | 0.20 |
| Yang 2016 ^(^[^41^](#_ENREF_41)^)^  Exp. 2 | Intervention diet with 5.2% LCMUFA-concentrate and 14.8% milk fat ^\|\| ¶^ | 1.81 | 0.892 | 0.010 | 0.021 | 0.073 | 0.18 |
|  | Comparator diet with 20% milk fat ^\|\| ¶^ | 0 | 0.024 | 0 | 0 | 0.073 | 0.19 |
| Yang 2017 ^(^[^42^](#_ENREF_42)^)^ | Intervention diet (5.1% CA-concentrate and 15.9% milk fat) ^\|\| ¶^ | 4.78 | 0.016 | 0 | 0 | 0.050 | 0.18 |
|  | Comparator diet (21% milk fat) ^\|\| ¶^ | 0 | 0.021 | 0 | 0 | 0.066 | 0.19 |
| Ostbye 2023 ^(^[^34^](#_ENREF_34)^)^ | Intervention diet (7% sandeel oil and 5% soybean oil) | 1.04 | N/A | 0.720 | 0.636 | 0.444 | 0.09 |
|  | Comparator diet (12% soybean oil) | 0 | N/A | 0 | 0 | 0.828 | 0 |

ALA; alpha-linolenic acid, CA; cetoleic acid, EPA; eicosapentaenoic acid, DHA; docosahexaenoic acid, GA; gadoleic acid, N/A, data not available

^*^ The article presented ΣC22:1 and ΣC20:1 and not individual isomers of C22:1 and C20:1

^†^ The fatty acid composition of the corn oil was not presented in this article, therefore data was taken from https://fdc.nal.usda.gov/fdc-app.html#/food-details/748323/nutrients and shows ΣC22:1 and ΣC20:1 and not individual isomers of C22:1 and C20:1

^‡^ The fatty acid composition of the olive oil was not presented in this article, therefore data was taken from https://fdc.nal.usda.gov/fdc-app.html#/food-details/1103861/nutrients and shows ΣC22:1 and ΣC20:1 and not individual isomers of C22:1 and C20:1

^§^ The fatty acid composition of the for soybean oil was not presented in this article, therefore data was taken from Yang 2013 ^(^[^36^](#_ENREF_36)^)^

^||^ The fatty acid composition of the milk fat was not presented in this article, therefore data was taken from https://fdc.nal.usda.gov/fdc-app.html#/food-details/173430/nutrients and shows ΣC22:1 and ΣC20:1 and not individual isomers of C22:1 and C20:1

^¶^ Both the intervention diet and the comparator diet were added 0.15% cholesterol

^**^ The cholesterol content of the fat sources was not presented in any of the articles, therefore the dietary cholesterol content was estimated based on information taken from https://fdc.nal.usda.gov/
